# Systematic review of global hepatitis E outbreaks to inform response and coordination initiatives

**DOI:** 10.1186/s12889-023-15792-8

**Published:** 2023-06-12

**Authors:** Fatima H. Al-Shimari, Camerin A. Rencken, Carl D. Kirkwood, Ramya Kumar, Kirsten S. Vannice, Barclay T. Stewart

**Affiliations:** 1 Department of Global Health, University of Washington School of Public Health, Seattle, WA USA; 2 Strategic Analysis, Research and Training (START) Center, Seattle, WA USA; 3 Department of Epidemiology, University of Washington School of Public Health, Seattle, WA USA; 4 Enteric and Diarrheal Diseases, Global Health, Bill & Melinda Gates Foundation, Seattle, WA USA; 5 Department of Surgery, University of Washington, Seattle, WA USA; 6 Harborview Injury Prevention and Research Center, Seattle, WA USA

**Keywords:** Viral Hepatitis, Hepatitis E, Vaccine, Outbreak, HEV

## Abstract

**Introduction:**

Hepatitis E virus (HEV) is the most common cause of acute hepatitis. While symptoms are generally mild and resolve within weeks, some populations (e.g., pregnant women, immunocompromised adults) are at high-risk of severe HEV-related morbidity and mortality. There has not been a recent comprehensive review of contemporary HEV outbreaks, which limits the validity of current disease burden estimates. Therefore, we aimed to characterize global HEV outbreaks and describe data gaps to inform HEV outbreak prevention and response initiatives.

**Methods:**

We performed a systematic review of peer-reviewed (PubMed, Embase) and gray literature (ProMED) to identify reports of outbreaks published between 2011 and 2022. We included (1) reports with ≥ 5 cases of HEV, and/or (2) reports with 1.5 times the baseline incidence of HEV in a specific population, and (3) all reports with suspected (e.g., clinical case definition) or confirmed (e.g., ELISA or PCR test) cases if they met criterium 1 and/or 2. We describe key outbreak epidemiological, prevention and response characteristics and major data gaps.

**Results:**

We identified 907 records from PubMed, 468 from Embase, and 247 from ProMED. We screened 1,362 potentially relevant records after deduplication. Seventy-one reports were synthesized, representing 44 HEV outbreaks in 19 countries. The populations at risk, case fatalities, and outbreak durations were not reported in 66% of outbreak reports. No reports described using HEV vaccines. Reported intervention efforts included improving sanitation and hygiene, contact tracing/case surveillance, chlorinating boreholes, and advising residents to boil water. Commonly missing data elements included specific case definitions used, testing strategy and methods, seroprevalence, impacts of interventions, and outbreak response costs. Approximately 20% of HEV outbreaks we found were not published in the peer-reviewed literature.

**Conclusion:**

HEV represents a significant public health problem. Unfortunately, extensive data shortages and a lack of standardized reporting make it difficult to estimate the HEV disease burden accurately and to implement effective prevention and response activities. Our study has identified major gaps to guide future studies and outbreak reporting systems. Our results support the development of standardized reporting procedures/platforms for HEV outbreaks to ensure accurate and timely data distribution, including active and passive coordinated surveillance systems, particularly among high-risk populations.

**Supplementary Information:**

The online version contains supplementary material available at 10.1186/s12889-023-15792-8.

## Introduction

Hepatitis E is a disease of the liver caused by infection with the hepatitis E virus (HEV), a non-enveloped single-stranded RNA virus [1, 2]. HEV isolates from different mammalian hosts are classified into four main groups, referred to as genotypes 1, 2, 3, and 4. Genotypes 1 and 2 affect only humans, while genotypes 3 and 4 have a wider range of hosts, causing infections in various mammalian species and sometimes spreading to humans [3]. The distribution of genotypes varies depending on geographic region. Genotypes 1 and 2 are most common among people who live in low- and middle-income countries (LMIC) [1, 4]. The aforementioned genotypes are primarily transmitted via the fecal-oral route, spreading through water pollution and sewage leaks, and are particularly prevalent in densely populated communities and those without well-organized waste water management systems [2, 5]. Genotypes 3 and 4 are less common and are transmitted to humans more commonly in high-income countries (HIC) and primarily through zoonosis such as contact with contaminated swine or pork products [6].

HEV generally causes an acute and self-limited illness characterized by hepatocyte infection and liver dysfunction with low mortality rates, particularly in high-income settings [7]. The clinical syndrome of HEV usually last less than two weeks and symptoms include fatigue, poor appetite, stomach pain, nausea, and jaundice [7]. However, HEV infection can be more severe among pregnant, very young, and elderly patients [3, 8]. HEV can cause fulminant liver failure, and in rare cases, chronic hepatitis (e.g., ≥ 3 months of viremia) in immunocompromised patients. Pregnant women with HEV, particularly those in the second or third trimester, are at increased risk of acute liver failure, fetal loss, and death [8] Mortality estimates for pregnant women range from 5.1 to 31%, with a 200 to 300% increased risk of intrauterine fetal death [8–10]. While there is no specific treatment for acute hepatitis E infection in patients, existing treatment options aim to relieve symptoms such as nausea, vomiting, and fatigue [9]. Patients with severe acute hepatitis E may require hospitalization, where they can receive intravenous fluids to maintain hydration, and may require supportive care for liver function [10].

The first documented HEV outbreak occurred in India between 1955 and 1956 [11]. During that outbreak, at least 293,000 people were symptomatic [11]. However, it was not until 1980 that HEV was identified as the agent causing the outbreak [12]. Since then, HEV has been identified as one of the most frequent causes of acute viral hepatitis globally [3]. The most recent models estimate 20 million new cases of HEV occur each year, of which about 20% are symptomatic [1]. One global burden estimate suggests that there were more than 70,000 deaths and 3,000 stillbirths attributed to HEV in 2005 [13].

A recombinant vaccine (e.g., Hecolin®, HEV239, Xiamen Innovax Biotech, China) was developed to prevent HEV disease. The subunit recombinant vaccine contains a 239 bp region corresponding to amino acid residues 368–606 of the capsid protein of genotype 1 [14]. HEV239 is administered in three doses scheduled over six months. The vaccine has been studied in a phase III trial in China among more than 100,000 participants [14]. Over a 12-month period and after 30 days post-primary series, there were no serious adverse events, and the vaccine had an efficacy rate of 100% (95% CI: 72.1–100.0) [14]. The vaccine has been licensed for use in China since 2012. The World Health Organization (WHO) has recommended the vaccine as a component of outbreak response, including use among pregnant women [15, 16]. However, there is a lack of data on the vaccine’s efficacy against specific genotypes (other than genotype 4) and estimates of epidemic disease burden to support wide use of the vaccine. Quantifying the global disease burden will help assess the value of a HEV vaccination in an outbreak response, and the size of a vaccine stockpile that might be needed.

While it is well recognized that HEV incurs a significant global health burden due to both endemic and epidemic disease, it remains neglected with respect to public health awareness and outbreak response [5, 17]. HEV is under-reported and there is limited information on outbreaks and disease surveillance, which means that our current estimates of the HEV-related burdens of disease are likely gross underestimates [5]. To address these gaps, we performed a systematic review to consolidate HEV outbreak data from the past ten years using peer-reviewed and gray literature (2011–2022). By doing so, the findings might further our understanding of HEV outbreak epidemiology and inform future outbreak prevention and response strategies.

## Methods

### Literature search

We searched PubMed and Embase for peer-reviewed records of HEV outbreaks published between January 1, 2011, and November 30, 2022. The search was conducted using the Preferred Reporting Items for Systematic Reviews and Meta-Analyses (PRISMA) guidelines [18, 19]. Search terms, Mesh for PubMed and Emtree for Embase, included terms like “hepatitis e,” “outbreak,” “epidemic,” and “humans” (Appendix 1). We used an ancestry approach to identify other records that potentially met the inclusion criteria and contributed to the aim of this review, including the reports in Hakim et al. 2017 systematic review of HEV outbreaks [20, 21].

We searched the Program for Monitoring Emerging Diseases (ProMED) for related gray literature. Reports from ProMED were included to mitigate the time delays and underreporting of HEV outbreaks in the peer-reviewed literature. Using analogous terms and timeline to those used for the peer-reviewed literature search, we followed the WHO Rapid Review Guidelines to conduct our systematic search within ProMED [22].

After retrieving the initial search results, we imported them into Microsoft Excel 2019 to organize and deduplicate the studies. Specifically, we used Excel’s “Remove Duplicates” feature to identify and remove any duplicate studies that were retrieved from multiple sources. We also manually screened the studies to ensure that they met our inclusion criteria. To track the screening and selection process, we created a spreadsheet in Excel with columns for study title, authors, year of publication, study design, population characteristics, outcome measures, and inclusion/exclusion criteria. We recorded the results of each stage of screening in separate sheets within the same Excel workbook, allowing us to easily track the progress of the review and ensure that all studies were screened and selected according to our pre-specified criteria. We used Microsoft Excel’s built-in sorting and filtering functions to explore the data and identify patterns in the studies, such as differences in study design or outcome measures across populations. We also used Excel to generate descriptive statistics on included study characteristics, such as mean sample size or publication year.

### Study selection

We included all reports (after deduplication) that published original data on HEV outbreaks. Two reviewers (CR and FA) independently screened records for eligibility. Any discrepancies were resolved by a third reviewer (RK) and group discussion. The same arbitration methods were employed during full-text report review. Our inclusion criteria were as follows: (1) reports including five or more cases of HEV, and/or (2) reports with 1.5 times the baseline incidence of HEV in a specific population, and (3) all reports with suspected (e.g., clinical case definition) or confirmed cases (e.g., ELISA or PCR test) if they met criterium 1 and/or 2. We excluded case reports of fewer than five cases of HEV, reports published before 2011, reports that were not published in English, reports of cases occurring in only animals, and laboratory studies of HEV rather than outbreaks.

### Data extraction and synthesis

We assessed all HEV outbreaks from 2011 to 2022 as our primary outcome. We defined outbreaks as having five or more cases of HEV infections, or 1.5 times the baseline incidence in a HEV-endemic setting. Summary data were extracted from reports including: year of report publication, date of official outbreak declaration, laboratory methods used to confirm HEV infection, genotypes identified, number of people suspected and confirmed to have HEV, ages of cases, number of pregnant women infected, outbreak location, outbreak setting (e.g., rural, urban, camp settings, military facility, factory), outbreak point source, risk factors, co-infections, case fatality rate (total and by sub-populations), and whether a vaccine or other intervention was systematically used. Data extraction was performed by three reviewers in accordance with WHO Rapid Review Guidelines (FA, CR, and RK) and quality checks were performed on a randomly generated subset of the data (10% of reports) (Appendix 2). ProMED-mail reports were also included in this analysis, allowing us to capture even small datasets and personal reports from clinicians and researchers.

## Results

### Literature search results

**Fig. 1:**
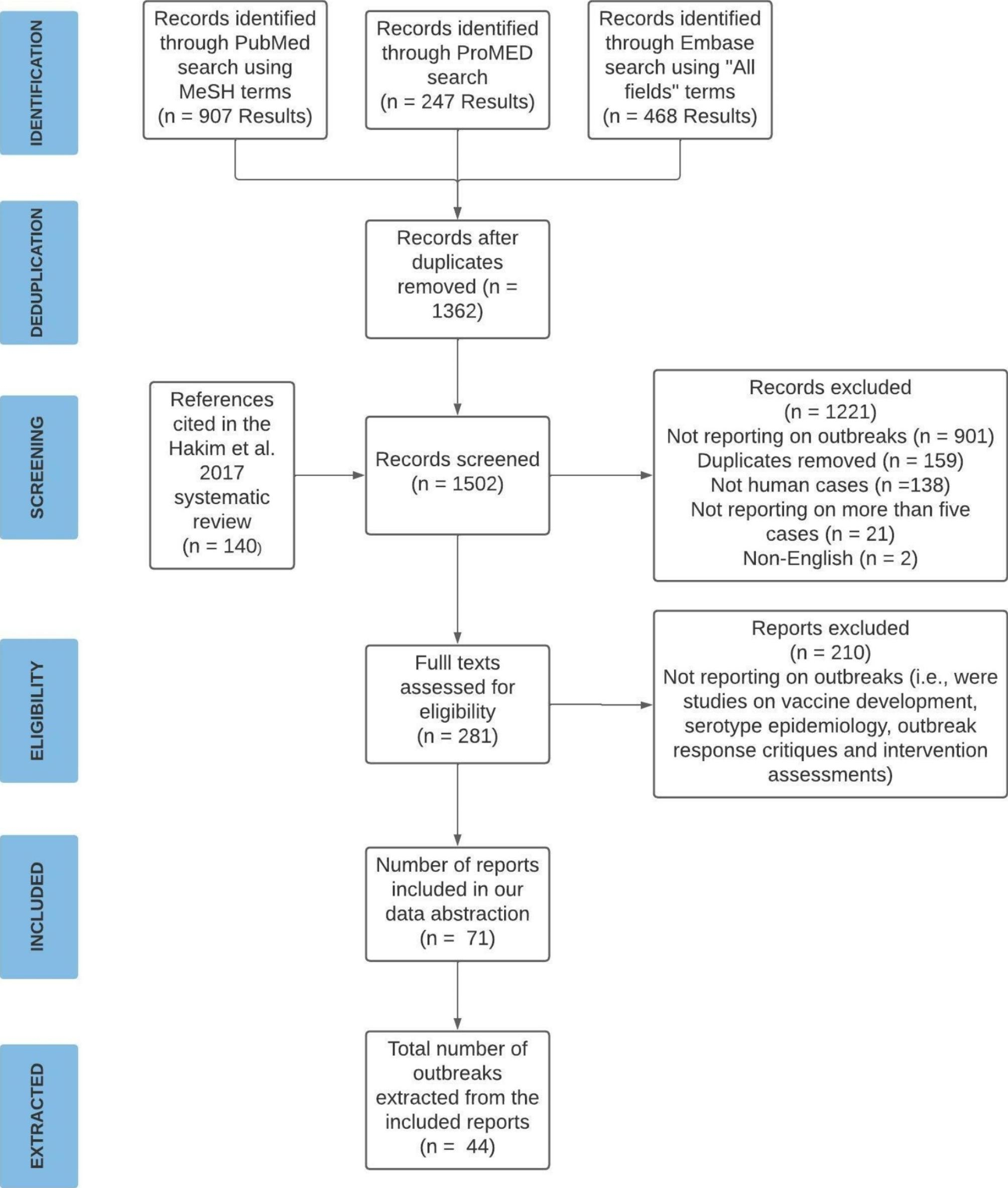
Preferred Reporting Items for Systematic Reviews and Meta-Analyses(PRISMA) Flow Diagram of Study Selection Process

Our search of peer-reviewed literature identified 907 records from PubMed and 468 from Embase (Fig. 1). We identified 247 ProMED records from the gray literature. In total, we screened 1,362 potentially relevant records after removing duplicates, and assessed 281 full-text reports for eligibility (Fig. 1). Seventy-one reports met our inclusion criteria for abstraction, of which 21 reports were from peer-reviewed literature (PubMed and Embase) and 50 reports were from gray literature (ProMED). These reports accounted for 44 outbreaks globally (Table 1; Appendix 2). Seventeen outbreaks were found in both the peer-reviewed and gray literature, seventeen were found in the peer-reviewed literature only, and ten were found in the gray literature only.

**Table 1:** Characteristics of Included Outbreak Reports (n = 44)

Outbreak Characteristic	n (%)
**Year of Outbreak Detection**	
2004–2006	1 (2.27%)
2007–2009	4 (9.09%)
2010–2012	9 (20.45%)
2013–2015	13 (29.55%)
2016–2018	13 (29.55%)
2019–2022	4 (9.09%)
**Geographic Region**	
African Region (AFR)	27 (61.36%)
South-East Asia Region (SEAR)	12 (27.27%)
European Region (EUR)	3 (6.82%)
Western Pacific Region (WPR)	2 (4.55%)
Eastern Mediterranean Region (EMR)	2 (4.55%)
Region of the Americas (AMR)	0 (0.0%)
**Outbreak Genotype**	
1	6 (13.64%)
2	2 (4.55%)
3	1 (2.27%)
4	3 (6.82%)
Did not report the HEV Genotype	32 (72.72%)
**Outbreak Detection Method**	
Jaundice	17 (38.64%)
Fever	6 (13.64%)
Pregnancy Screening	4 (9.09%)
Acute Liver Disease Symptoms	1 (2.27%)
Did not report how suspected cases were identified	16 (36.36%)
**Outbreak Lab Confirmation Method**	
Enzyme-linked immunosorbent assay (ELISA)	15 (34.09%)
Polymerase chain reaction (PCR)	9 (20.45%)
“Molecular characterization tests performed in Nairobi”	1 (2.27%)
Did not report whether lab confirmation was used	19 (43.18%)
**Outbreak Point Source**	
Waterborne Transmission	21 (47.72%)
Live animals or Food containing pork products	4 (9.09%)
Point source was not reported	19 (43.19%)

### Epidemiology

#### Location and settings

The 44 identified outbreaks occurred in 19 countries (Appendix 2). Most of the reports described outbreaks in Africa (n = 27, 61.4%) and Southeast Asia (n = 12, 27.3%) regions (Table 1). Humanitarian settings (e.g., camp settings such as refugee camps and internally displaced person settlements) were the most common places (n = 12, 27.3%) where HEV outbreaks were identified, followed by hospital (n = 7, 15.9%) and factory (workplace) settings (n = 2, 4.5%).

#### Risk factors and outbreak sources

Waterborne transmission was the most common point-source of the outbreaks, attributed to 21 (47.7%) of the outbreaks reported (Table 2). Fecal contamination of drinking water occurred through different mechanisms, including leakage from water pipelines due to faulty infrastructure, and a failure to treat water in communities with known contaminated water sources (e.g., formal wastewater management, household filtration, chlorination) (Table 2). One outbreak investigation found that cases were more common in regions with rainy seasons or floods because heavy rain and wind overwhelmed, and occasionally compromised the effectiveness of WASH facilities [23].

**Table 2:** Included Outbreak Characteristics Stratified by WHO Region

Outbreak Characteristic	AFR	AMR	EMR	EUR	SEAR	WPR
Number of outbreaks reported	27	0	2	3	10	2
Number of countries with these outbreaks	13	0	2	1	3	1
Number of confirmed cases (not including pregnant women) *	14,846	0	95	133	5,830	435

*These values are subject to under-reporting

WHO regions include the African Region (AFR), the Eastern Mediterranean Region (EMR), the South-East Asia Region (SEAR), the Region of the Americas (AMR), the Western Pacific Region (WPR), and the European Region (EUR)

The second most common source of outbreaks came from HEV transmission by live animals or food containing pork products. Four (9.1%) of the outbreaks were caused by genotype 4, and all took place in higher income countries from contaminated food sources. No outbreak identified a hospital or clinical settings as the initial point-source, although many outbreaks were detected in hospital settings and included health workers.

#### Case identification, testing, and genotypes

There was marked heterogeneity in how people were selected for HEV testing during outbreaks (Table 2). Some were considered for testing after displaying signs of HEV (e.g., jaundice) while others were tested because they lived in an area with contaminated water sources regardless of the presence or absence of clinical symptoms. The most frequently employed method of detecting potential or suspected HEV cases was through use of clinical criteria (e.g., clinical case definition). Seventeen outbreaks (38.64%) were identified by testing those with jaundice alone, six (13.64%) tested those with fevers, four (9.09%) tested pregnant women, one (2.27%) only tested those who displayed symptoms of acute liver disease (e.g., symptoms of jaundice, fatigue, with or without encephalopathy), and 16 (36.36%) reports did not describe how suspected outbreaks were identified.

Twenty-five of the 44 outbreaks (56.82%) were laboratory confirmed (e.g., in addition to use of a clinical case definition in a high-risk area or during a known outbreak). Enzyme-linked immunosorbent assay (ELISA) and polymerase chain reaction (PCR) were the most common methods of confirmation of HEV infection, where 15 (34.1%) outbreaks were confirmed by ELISA and 9 out of 44 (20.5%) were confirmed by PCR. There was no sensitivity or specificity reported from the two laboratory methods in any report. One outbreak (2.3%) was confirmed by “molecular characterization tests,” but did not specify the specific type of assay. Almost half of the outbreak reports (n = 19, 43.2%) did not report whether lab confirmation occurred.

While most reports (n = 32, 72.7%) did not characterize the genotype responsible for the outbreak, HEV genotypes 1 was attributed to 6 or 13.6% of outbreaks, and 4 was attributed to 3 or 6.8% of outbreaks (Table 2). The outbreaks caused by genotype 1 were detected in Africa (Chad and South Sudan), and Southeast Asia (Bangladesh and India) (Table 2). The outbreaks caused by genotype 4 were detected in Europe (Italy) and East Asia (China), all of which were attributed to pork products. In addition, in Africa two outbreaks (4.6%) were caused by genotype 2, [23, 24] and one outbreak was caused by genotype 3 [25]. There was not enough information reported for each outbreak to determine whether there were clinical or epidemiological differences in these outbreaks by genotype.

#### Age groups

There was significant heterogeneity in the reporting of age groups among HEV cases (Appendix 2). Age ranges of infected and/or symptomatic individuals were usually reported as aggregates in large intervals (e.g., 0–64 years, 8–65 years). Some reports provided the mean and standard deviation of age groups in addition to an age interval [e.g., 1–87 years (average age 27.9 years ± 5.1)], thus we were unable to further disaggregate the age distribution of HEV cases [26].

#### Case descriptions, high-risk groups, and fatalities

High-risk groups for infection included people with pre-existing liver disease and/or jaundice, people living in camp settings with limited WASH facilities, and pregnant women. Jaundice was mentioned as the most common symptom for HEV cases, identified in 61% of patients. Other outbreaks only reported related signs and symptoms such as dark urine, elevated serum transaminases and overt liver failure (e.g., hypoglycemia, coagulopathy, encephalopathy).

Case fatalities per outbreak ranged from 0.22% in an outbreak in rural India [9] (9 fatalities reported out of 4,085 confirmed cases) to 22.8% in a refugee camp within South Sudan [27] (101 fatalities reported out of 443 confirmed cases). Among the documented case fatalities, the proportion of pregnant women who died from these outbreaks ranged from 17.9% in a refugee camp in South Sudan [27] (22 out of 123 total fatalities) to 44.7% in a refugee camp in Niger (17 out of 38 total fatalities).

### Outbreak sizes and durations

Most of the reports did not indicate the number of people at risk, although some did indicate a population size (e.g., number of people living in a town, camp, or region proximate to an outbreak). The number of confirmed cases per outbreak ranged from 5 to 4,085 (not including pregnant women) and confirmed cases among pregnant women ranged from 3 (out of 278 suspected cases) to 211 (out of 576 suspected cases). The duration of the outbreaks varied from 4 weeks to 3 years, while 36% of outbreaks (16 out of 44) did not report the duration [24, 27]

### Outbreak response initiatives and opportunities

Few reports described specific outbreak responses and public health interventions undertaken. The most commonly reported activities included enhanced case surveillance [30] (e.g., passive syndromic surveillance at health facilities, active community-based surveillance using case definitions), targeted prevention efforts [39] (e.g., distribution of hygiene kits containing bars of soap and buckets, dissemination of water filtration or purification supplies), and supportive case management to prevent fatalities (Table 3).

**Table 3:** Multilateral Interventions to Prevent, Detect, and Contain Hepatitis E Outbreaks

System Level	Prevention	Detection	Response
Local	- Raise awareness about HEV and mobilize communities to get screened - Promote partnerships across public health services maintain updated health records	- Offer HEV testing kits to local community members and encourage communities to report symptoms or positive test results to local healthcare facilities	- Report HEV cases to national authorities and maintain surveillance of existing HEV cases
National	- Maintain quality standards for public water supplies - Establish proper disposal systems for human fecal matter	- Enhance screening and testing sites for HEV - Fund healthcare facilities to enhance their capacity to admit and treat HEV patients	- Scale up screening, care, and treatment services - Alert the public and the WHO about HEV outbreaks
Regional	- Collaborate with national governments to establish universal HEV screening programs - Establish a case definition for HEV and promote its use across WHO regions	- Formulate evidence-based policy and data for action - Promote partnerships across laboratories and healthcare facilities in the region	- Supply RDT to confirm cases and keep an accurate record of case morbidities and mortalities
World Health Organization (WHO)	- Connect with national governments to underscore the value of alerting regional offices about suspected or confirmed HEV outbreaks - Establish a universal standard for the minimum level of data to be collected for HEV cases and outbreaks	- Supply Rapid Diagnostic Tests (RDT) to confirm cases	- Establish and maintain an open-source database for countries to document HEV outbreaks - Supply RDT to confirm cases and keep an accurate record of case morbidities and mortalities

### Data gaps and outbreak identification

The findings highlighted several significant reporting gaps. Many outbreaks (n = 27, 61.4%) did not mention the total number of HEV cases, case fatalities, or a population at risk. Additionally, the number of confirmed cases among pregnant women was not always reported (n = 30, 68.2%). Furthermore, there were gaps in whether the outbreaks were officially declared to have begun and ended, and when those dates were. Of the included reports, 31 (70.5%) did not mention that the outbreak was officially declared by local or national public health authorities, and 35 (79.5%) had no concluding outbreak report. Finally, 40 reports (90.9%) did not mention whether the HEV vaccine was used to prevent further transmission during the outbreak or prevent future outbreaks among high-risk populations (e.g., internally displaced people or refugees, pregnant women, people living in areas with prior outbreaks). Three reports mentioned the existence of the HEV vaccine; however, the vaccine had not been used in the respective outbreak suggesting a know-do gap: the gap between what we know and what we do in practice. Other commonly missing key data elements included specific case definitions used, testing strategy and method, impact of interventions, and costs of response. There was no central reporting platform to support standardized data collection and response.

## Discussion

The global burden of HEV is under-reported, in part, due to a lack of information about the epidemiology of HEV outbreaks [5, 17]. In recent years, however, awareness of its impact has become more widely recognized. Public health problems associated with HEV are particularly prevalent in LMICs, which have a lack of resources to respond to outbreaks and more people living in humanitarian settings. It is important to estimate the clinical disease accurately, especially among vulnerable populations such as pregnant women and refugees who are at a higher risk of severe morbidity and mortality. Identifying knowledge gaps around HEV outbreaks will allow future studies to fill in this information gap and assist in developing strategies to reduce the burden of this preventable disease worldwide.

Most of the reported outbreaks occurred in camp settings [5, 10, 28, 31] with waterborne transmission as their primary mode of transmission. Our findings are consistent with an older review of HEV outbreaks in sub-Saharan Africa that found that 50% of the outbreaks occurred in camp settings (e.g., refugee camps or internally displaced person (IDP) camps) [5]. These camps were typically the result of conflict and complex humanitarian emergencies rather than natural disaster [21, 31]. The density of the resident population, limited access to safe drinking water, and lack of adequate sanitation predispose individuals in these settings to the transmission of HEV. Due to the higher risk of HEV outbreaks in camp settings, it is important to implement passive and active surveillance systems, as well as promote hygiene and distribute home-based water purification supplies. In addition, vaccinating people living in IDP camps against HEV can significantly reduce both their morbidity and mortality, as well as prevent wider outbreaks. This is particularly crucial since many displaced people eventually settle in densely populated urban areas after leaving the camps, as is the case in countries like Syria, Afghanistan, Yemen, Ukraine, Ethiopia, and Nigeria.

Additionally, outbreak preparedness activities should be prioritized to maximize timeliness and effectiveness of response and coordination, including standard case definition, clinical training, surge response planning, implementation of national, regional, and global reporting schemes, and wider prevention interventions to limit outbreak size and duration. Risk factors for higher mortality rates with HEV infection, such as malnutrition, unsafe living conditions, and poor health related to living conditions, are found within displaced populations at a higher prevalence when compared to the general populations and should be incorporated into prevention and response planning initiatives [10]. In addition to people living in camp settings, pregnant women are at a particularly high risk of HEV infection and illness [21]; research has shown that pregnant women are more vulnerable to HEV than other viral hepatitides [8]. One report found that death during pregnancy increased 700% with HEV infection [8]. While we did not have conclusive data on pregnancy-related mortality rates during outbreaks, relative to the general public, the literature is clear that mortality rates among pregnant women were higher [21]. Consideration of comprehensive WASH initiatives and vaccination, with particular social mobilization around pregnant women, should be a priority.

Commonly reported interventions for outbreak prevention and control included improving sanitation and hygiene, advising residents to boil water, contact tracing and case surveillance, and chlorinating boreholes. During one outbreak, a humanitarian aid organization, Médecins Sans Frontières, distributed over 11,000 bars of soap and buckets to improve hygiene and lower the risk of HEV infection [29]. No impact evaluation was reported. The findings from this review are in line with the review conducted by Hakim et al. that found that water chlorination, improving hygiene, providing a safe water supply, and improving human waste disposal were all useful outbreak intervention strategies and useful even in refugee/IDP contexts [21]. However, no other innovative strategies or vaccination campaigns were described.

One key finding in this study was the lack of not only vaccine use, but the lack of planning for the potential use of the vaccine to prevent or control outbreaks. Until recently, Hecolin had never been deployed for outbreak response, so no vaccine feasibility or effectiveness estimates were available from an outbreak context. Three reports discussed its potential routine use. One report noted that while the vaccine has been approved, they were unable to implement it as part of a comprehensive outbreak response, due to “insufficient safety and efficacy data.” [23] While safety and efficacy data outside China are currently limited, there are ongoing trials to address this concern such as the phase IV cluster-randomized vaccine trial in Bangladesh among 20,000 women of childbearing age to evaluate their protection from HEV and identity risk factors for severe infection [32]. A Ph2b study in pregnant women has commenced in Pakistan, while an age de-escalation and safety study is in planning stages in South Africa. Additionally, the vaccine is registered in Pakistan and a clinical trial is ongoing in the United States. The recent catastrophic flooding and displacement of more than 3 million people in Pakistan make its registration and potential use to prevent HEV particularly timely and critical [33].

Our study findings indicated that non-standardized criteria were often used to define suspected cases during HEV outbreaks. Some reports only considered physical signs and/or symptoms [31] (e.g., jaundice, fatigue), whereas others required laboratory-confirmed methods (e.g., ELISA, PCR). Coordination among governments and public health agencies to increase availability of rapid HEV detection tests (RDTs) especially for use in LMICs, refugee/IDP contexts, and endemic settings may help estimate the true burden of HEV outbreaks and facilitate more robust responses. The WHO, affected governments, and humanitarian actors should consider developing a toolkit for the diagnosis, triage, and management of HEV cases during an outbreak, similar to the successful toolkit developed for HIV testing or Ebola virus disease (EVD) response [34].

Importantly, these findings highlight significant gaps in outbreak reporting. More than half of the outbreak reports were missing key data elements required for defining the epidemiology and planning an effective response for that outbreak as well as future ones. HEV case fatalities in outbreaks reported in the literature are significant underestimates because there are protracted outbreaks, insufficient surveillance systems, no standard of lack of standardized/centralized reporting platforms. Without systematically collected and reported data, efforts are hindered in their ability to not only respond to the outbreak but develop appropriate preventive measures, and plan better responses to future outbreaks. Lessons from centralized reporting platforms used during EVD and COVID-19 outbreaks regarding the development, implementation, and use of such platforms are potentially useful [35]. Key stakeholder consensus on a minimum dataset, accountability framework and data platform for reporting outbreaks, should be prioritized. Additionally, given the immense burden of HEV, we suggest implementation of both active and passive coordinated surveillance systems in high-risk settings.

Lastly, it is important to note that eight of the outbreaks reported no information in the peer-reviewed literature and therefore likely would have been missed if we had not searched gray literature. Additionally, it is possible that publication bias influenced our results given that humanitarian actors are more likely to be present in refugee/IDP settings than responding to outbreaks in countries with functioning health systems. This emphasizes the need for a global, publicly available data platform to consolidate all known information about HEV outbreaks, as well as a universal protocol for detecting, reporting, and responding to HEV outbreaks (Table 3). The emergence of influenza A (H1N1) mobilized the WHO to update the International Health Regulations (IHR) to require that all WHO Member States meet minimum standards for detecting, reporting, and responding to pandemics [36]. The revised IHR framework enabled a more coordinated global response to the 2009 influenza pandemic and more recent COVID-19 pandemic, because countries were reporting cases early and with enough information [36].

## Limitations

Our study has several limitations worth consideration. There are known gaps between outbreak response and public reporting processes and expectations. Outbreaks that occurred prior to the search date should not be interpreted as complete, as those publications may have occurred earlier. However, by systematically searching both peer-reviewed literature and ProMED, we were able to consolidate data available regarding contemporary HEV outbreaks. WHO has recognized the value of non-governmental organizations and the media in reporting outbreaks [37, 38]. By including ProMED-mail reports in this analysis, we were able to capture small datasets and personal reports from clinicians and researchers reporting directly from the outbreak. The ProMED system allows such reports to be published much more quickly and with less detail than what is typically required for journal publications. Outbreak reports has a lot of information missing. Second, the search was not designed to capture reports of HEV vaccine use in endemic settings. However, there was also no reported use among outbreak reports from locations with endemic HEV. Lastly, synthesis of our findings and the recommendations for actionable response are limited by the heterogeneity of data elements reported. However, this itself is a key finding that must be addressed if we are committed to characterizing and reducing the burden of HEV.

## Conclusions

This study aimed to describe available data about HEV outbreaks to inform response and control initiatives. HEV represents a significant public health problem, especially in LMICs that have limited resources to respond to outbreaks and greater numbers of people living in high-risk, humanitarian settings, such as camps. Accurate estimates of the clinical disease are needed, particularly among vulnerable populations such as pregnant women or refugees who are at an increased risk of severe morbidity and mortality. By identifying gaps in knowledge around HEV outbreaks, we hope that future studies can begin to fill in this missing data and decrease the burden of this preventable disease worldwide.

## Electronic supplementary material

Below is the link to the electronic supplementary material.

Supplementary Material 1

Supplementary Material 2

## Data Availability

Data supporting the results reported in the article can be found in the “Appendix 2. Outbreak Reports” table.

## List of Abbreviations

ELISA: Enzyme-linked immunosorbent assay
HEV: Hepatitis E virus
HIC: High-income countries
LMIC: Low- and middle-income countries
PCR: Polymerase chain reaction
RDT: Rapid detection tests
WHO: World Health Organization
