# Supplementary material for: Systematic review of global hepatitis E outbreaks to inform response and coordination initiatives
Source: BMC Public Health. 2023 Jun 12;23:1120. doi: 10.1186/s12889-023-15792-8 (PMC10259355; doi:10.1186/s12889-023-15792-8)
Supplement: Supplementary file 1 — Supplementary Material 1 [file 12889_2023_15792_MOESM1_ESM.docx]

**Appendix 1. Search Strings**

| **PubMed** |
| --- |
| ("Hepatitis E"[Mesh] OR "Hepatitis E virus"[Mesh] OR "hepatitis e" or "water-borne hepatitis") AND (("Disease Outbreaks"[Mesh]) or "disease outbreak" or "Epidemics"[Mesh] or epidemic or prevalence) NOT (comment(PT) or editorial(PT) or letter (PT)) AND (2015:2021[pdat]) AND (humans[Filter]) AND (English[Language]) |
| **Embase** |
| ('hepatitis e' OR 'hepatitis e virus' OR 'hepatitis e vaccine’) AND (outbreak OR epidemic) AND English AND human |
| **ProMED** |
| “Hepatitis E” “Hepatitis E Virus” “HEV” “HEV outbreaks” |

**Appendix 2. Outbreak Reports**

| Year Outbreak was Detected | Outbreak Country | No. Confirmed Cases | HEV Genotype | Age range of Affected Population | Population Demographic | Outbreak Duration | Link: | Number of ProMed Reports |
| --- | --- | --- | --- | --- | --- | --- | --- | --- |
| 2004-2005 | Bangui, Central African Republic | 411 | Not Reported | 1-87 years (average age, 27.9 ± 5.1); study aggregated ages as 1-17, 18-33, 34+ | Patients at one of 11 health care centers in the Central African Republic that displayed symptoms of HEV | Not Reported | <https://www.ncbi.nlm.nih.gov/pmc/articles/PMC3089785/> | Supplemented information from <https://reliefweb.int/report/central-african-republic/central-african-republic-hepatitis-e-outbreak-capital> |
| 2007 | Karachi, South Pakistan | 53 (81.5%) were positive for HEV RNA | Genotype 1 | Ages not specified in the study; for 65 patients with Acute Liver Disease, the median age was 30.9 years | All patients attending the outpatient and inpatient clinics of The Aga Khan University, between December 1, 2007, and July 31, 2008 | Not Reported | <https://pubmed.ncbi.nlm.nih.gov/22938992/> | N/A |
| 2007-2008 | Giza, Egypt | 42 | Not Reported | 2-54, aggregated | Kom El-Mansoura villagers | Not Reported | <https://bmcinfectdis.biomedcentral.com/articles/10.1186/s12879-020-04961-4> | N/A |
| 2008 | Central African Republic | 745 | 1e and 2b | Mean and IQR (23, 17–33) | Patients with jaundice | 12 months | <https://pubmed.ncbi.nlm.nih.gov/25092862/> | N/A |
| 2009-2012 | Karamoja, Uganda | 987 | Genotype 2 | 0-60+, data disaggregated by 10-year clusters | Semi-nomadic pastoralist population | prolonged, lasted 3 years | <https://www.ncbi.nlm.nih.gov/pmc/articles/PMC4001480/> | N/A |
| 2010 | Bangladesh | 48 | 1 | 8-65 years, with a mean of 31 and a median of 28 years | Patients with jaundice | 4 months | <https://promedmail.org/promed-post/?id=753757> | 1 |
| 2010-2011 | Sudan | 39 | Not Reported | The mean (SD) maternal age and gestational age were 24.0 (4.2) years and 33.6 (3.7) weeks, respectively | Pregnant women at Port Sudan Hospital, Eastern Sudan | 4 months | <https://promedmail.org/promed-post/?id=989539> | 1 |
| 2011 | Italy | 81 | 41/89 cases were viremic (Genotypes 1,2,3, and 4) | Mean age reported: 55.5 ± 16.7 | Patients with immune-suppression, chronic liver disease, acute liver failure, and acute kidney injury | Not Reported, but data was collected from 2011-2018 | <https://www.ncbi.nlm.nih.gov/pmc/articles/PMC4604969/> | N/A |
| 2011 | Lazio, Italy | 5 | Genotype 4 | 38-74, five patients with disaggregated data | A population of 5 Males in Lazio | Not Reported | <https://promedmail.org/promed-post/?id=1295615> | 6 |
| 2011 | Karamoja, Uganda | 908 | Not Reported | Not Reported | Not Reported | Not Reported | <https://promedmail.org/promed-post/?id=1735825> | 5 |
| 2011 | Karamoja, Uganda | at least 68 | Not Reported | Not Reported | Not Reported | Approximately 2 months | <https://promedmail.org/promed-post/?id=2115912> | 1 |
| 2012 | Maban County, Upper Nile State, South Sudan | 443 | Genotype 1 | 6-64 years | Refugees | 6 months | <https://promedmail.org/promed-post/?id=2157139> | 2 |
| 2012 | South Sudan | Not Reported | Not Reported | Median age 25 years | People living in a refugee camp | Not Reported | <https://promedmail.org/promed-post/?id=1822306> | 1 |
| 2012 | Kolhapur, India | 4085 | 1 | Not Reported | Not Reported | Not Reported | <https://www.researchgate.net/publication/275961161_First_documented_outbreak_of_Hepatitis_E_in_Northern_Cameroon_Demanou_Maurice_Mahamat_Abassora_Nimpa_M_Marcelin_Njouom_Richard_Annals_of_Tropical_Medicine_and_Public_Health_Year_2013_Volume_6_Issue_6_> | N/A |
| 2013 | China | 394 | 4 | 0-59 years | Not Reported | The disease occurred sporadically throughout the year. The peak value of reported patients appeared in January, February and/or March from 2011 to 2016. | <https://promedmail.org/promed-post/?id=2676571> | 1 |
| 2013 | Sudan | 2572 | Not Reported | Not Reported | People living in a refugee camp | Not Reported | <https://promedmail.org/promed-post/?id=3636631> | 2 |
| 2013 | Tanzania | Not Reported | Not Reported | Approximately 61% of reported cases occurred in individuals below the age of 15 years | Not Reported | 2 months | <https://promedmail.org/promed-post/?id=4405317> | 1 |
| 2013 | Karamoja, Uganda | 1000 | Not Reported | Not Reported | sub-counties in Karamoja | Not Reported | <https://promedmail.org/promed-post/?id=4126018> | 1 |
| 2013 | Moroto, Uganda | 40 | Not Reported | Not Reported | Not Reported | Not Reported | <https://promedmail.org/promed-post/?id=4791758> | 3 |
| 2013 | Cameroon and Chad | 37 | This is to the best of the researchers' knowledge, the first ever documented outbreak of hepatitis E in Cameroon despite multiple outbreaks reported in the neighboring Chad during the last 2 decades. | 7-50 years, with 21% of those cases between 0-14 years | The local health personnel suspected yellow fever virus infection and blood sample collected were sent to the National Reference Laboratory for yellow fever at Centre Pasteur of Cameroon (CPC) for confirmation. | Residents residing in two neighboring villages (Bogdibo and Golonbali) situated along the Cameroon-Chad border | <https://www.ncbi.nlm.nih.gov/pmc/articles/PMC5703542/> | N/A |
| 2014 | India | 84 | 1a | 2–65 years | Pregnant women and persons with AJS | As per records, jaundice was reported in sporadic cases since September 2014 and suddenly the numbers increased in end November–early December 2014; the last jaundice case was seen on 7 February 2015 and the outbreak was declared over | <https://promedmail.org/promed-post/?id=5455477> | 5 |
| 2014 | Ethiopia | 367 | Not Reported | Not Reported | People living in a refugee camp | Not Reported | <https://promedmail.org/promed-post/?id=5393925> | 7 |
| 2014 | Nepal | "6000 afflicted" | Not Reported | 15–40 years | Inmates, townspeople | Not Reported | <https://promedmail.org/promed-post/?id=5393925> | 1 |
| 2014 | Patna, India | Not Reported | Not Reported | Not Reported | Not Reported | Not Reported | <https://promedmail.org/promed-post/?id=5522167> | 18 |
| 2014 | Kangra, India | 818 | Not Reported | Health authorities identified 55 patients including children below the age of 15 years | Not Reported | Not Reported | <https://www.cdc.gov/mmwr/volumes/69/wr/mm6912a6.htm?s_cid=mm6912a6_w> | N/A |
| 2014 | Sambalpur, India | Not Reported | Not Reported | Not Reported | Residents of Sambalpur | Approximately 2 months | <https://promedmail.org/promed-post/?id=8557918> | 1 |
| 2015 | South Sudan | 696 | Not Reported | Not Reported | Not Reported | Jun-14 | <https://promedmail.org/promed-post/?id=8605063> | 1 |
| 2016 | Sudan | Not Reported | Not Reported | Not Reported | Not Reported | May-16 | <https://promedmail.org/promed-post/?id=4405317> | 1 |
| 2016 | Kenya | 21 | Not Reported | Not Reported | Not Reported | Not Reported | <https://www.ncbi.nlm.nih.gov/pmc/articles/PMC3089785/> | Supplemented information from <https://reliefweb.int/report/central-african-republic/central-african-republic-hepatitis-e-outbreak-capital> |
| 2016 | India | 1600 | Not Reported | Not Reported | Not Reported | Jan 2016 - Mar 2016 | <https://pubmed.ncbi.nlm.nih.gov/22938992/> | N/A |
| 2016 -2017 | Chad | 956 | Not Reported | Not Reported | Not Reported | August 2016 - Feb 2017 | <https://bmcinfectdis.biomedcentral.com/articles/10.1186/s12879-020-04961-4> | N/A |
| 2017 | Chad | 100 | 1e | 15–44 years | Pregnant women, children <1 year of age and persons with AJS who were vomiting and/or had altered mental status | 44 weeks | <https://pubmed.ncbi.nlm.nih.gov/25092862/> | N/A |
| 2017 | Nigeria | Not Reported | Not Reported | Not Reported | Internally Displaced Persons | Feb 2017 to Nov 2017 | <https://www.ncbi.nlm.nih.gov/pmc/articles/PMC4001480/> | N/A |
| 2017 | Niger | 1987 | Not Reported | Not Reported | Pregnant women and people aged 15 and over were most affected | Since April 2017 | <https://promedmail.org/promed-post/?id=753757> | 1 |
| 2017 | Niger | 736 | Not Reported | <5: 12 (1.6%), 5-14: 54 (7.3%), 15-24: 181 (24.6%), 25-34: 318 (43.2%), >35: 171 (23.2%) | People living in a refugee camp | Ongoing in this report | <https://promedmail.org/promed-post/?id=989539> | 1 |
| 2017 - 2020 | Namibia | 7457 | Not Reported | Not Reported | Not Reported | Dec 2017 - March 2020 | <https://www.ncbi.nlm.nih.gov/pmc/articles/PMC4604969/> | N/A |
| 2017-2020 | Namibia | During December 14, 2017–February 2, 2020, a total of 7,247 outbreak-associated hepatitis E cases were reported | This is the third hepatitis E outbreak described in Namibia since 1983, the largest to date, and the first of nationwide scope | 72% of cases were between ages 20–39 years | 2,677 (37%) cases were reported from three of Namibia’s largest informal settlements, Havana and Goreangab (both in Khomas region), and the Democratic Resettlement Community (in Erongo region) | Not Reported | <https://promedmail.org/promed-post/?id=1295615> | 6 |
| 2018 | China | 41 | 4 | 20-59 years | 302 of the 330 workers in a mechanical factory in Qingdao | 4 weeks | <https://promedmail.org/promed-post/?id=1735825> | 5 |
| 2018 | Bangladesh | Not Reported | Not Reported | Not Reported | Not Reported | April 2018 - | <https://promedmail.org/promed-post/?id=2115912> | 1 |
| 2018 | India | Not Reported | Not Reported | Not Reported | Not Reported | March 2018 to October 2018 | <https://promedmail.org/promed-post/?id=2157139> | 2 |
| 2019 | Italy | 47 | 3e and 3f | 28–85 years; median age was 63 years | Not Reported | 24 weeks | <https://promedmail.org/promed-post/?id=1822306> | 1 |
| 2019 | India | 32 | Not Reported | Of the total 49 suspected subjects, the mean ± SD age patients was 33.7 ± 13.8 years (IQR, 24.5-38.5 years); most of the individuals were between 10-25 years | Workers, farmers, students, and housewives | Ongoing in this report | <https://www.researchgate.net/publication/275961161_First_documented_outbreak_of_Hepatitis_E_in_Northern_Cameroon_Demanou_Maurice_Mahamat_Abassora_Nimpa_M_Marcelin_Njouom_Richard_Annals_of_Tropical_Medicine_and_Public_Health_Year_2013_Volume_6_Issue_6_> | N/A |
| 2021 | Sudan | Not Reported | Not Reported | Not Reported | Not Reported | Not Reported | <https://promedmail.org/promed-post/?id=2676571> | 1 |
| 2021 | South Sudan | 905 | Not Reported | Not Reported | Not Reported | Ongoing - since 2019 | <https://promedmail.org/promed-post/?id=3636631> | 2 |
