# Supplementary material for: Systematic review of global hepatitis E outbreaks to inform response and coordination initiatives
Source: BMC Public Health. 2023 Jun 12;23:1120. doi: 10.1186/s12889-023-15792-8 (PMC10259355; doi:10.1186/s12889-023-15792-8)
Supplement: Supplementary file 2 — Supplementary Material 2 [file 12889_2023_15792_MOESM2_ESM.docx]

**Protocol for a Systematic Review on Hepatitis E Virus Outbreaks: A Literature Search and Deduplication Method Using Microsoft Excel**

**Background:**

Hepatitis E virus (HEV) outbreaks were a significant public health concern, and a systematic review was needed to analyze the available data on HEV outbreaks. The aim of this review was to provide a comprehensive overview of HEV outbreaks that occurred between January 1, 2011, and November 30, 2022. In this protocol, we described the methods used for the literature search and deduplication of studies.

**Methods:**

A systematic review of the literature was conducted using the Preferred Reporting Items for Systematic Reviews and Meta-Analyses (PRISMA) guidelines. PubMed and Embase databases were searched for peer-reviewed records of HEV outbreaks published between January 1, 2011, and November 30, 2022. Additionally, the Program for Monitoring Emerging Diseases (ProMED) was searched for related gray literature. All reports (after deduplication) that published original data on HEV outbreaks were included.

The initial search results were imported into Microsoft Excel 2019 to organize and deduplicate the studies. Excel's "Remove Duplicates" feature was used to identify and remove any duplicate studies retrieved from multiple sources. The studies were also manually screened to ensure they met our inclusion criteria. The screening and selection process was tracked using a spreadsheet in Excel with columns for study title, authors, year of publication, study design, population characteristics, outcome measures, and inclusion/exclusion criteria.

Two reviewers independently screened records for eligibility, and any discrepancies were resolved by a third reviewer. Inclusion criteria were reports including five or more cases of HEV or reports with 1.5 times the baseline incidence of HEV in a specific population. Case reports of fewer than five cases of HEV, reports published before 2011, reports not published in English, reports of cases occurring in only animals, and laboratory studies of HEV rather than outbreaks were excluded.

Summary data was extracted from reports including year of report publication, date of official outbreak declaration, laboratory methods used to confirm HEV infection, genotypes identified, number of people suspected and confirmed to have HEV, ages of cases, number of pregnant women infected, outbreak location, outbreak setting (e.g., rural, urban, camp settings, military facility, factory), outbreak point source, risk factors, co-infections, case fatality rate (total and by sub-populations), and whether a vaccine or other intervention was systematically used. Data extraction was performed by three reviewers in accordance with WHO Rapid Review Guidelines, and quality checks were performed on a randomly generated subset of the data.

**Conclusion:**

This protocol outlined the methods for conducting a systematic review on HEV outbreaks. By using PRISMA guidelines and Microsoft Excel for deduplication, screening, and data organization, a comprehensive overview of HEV outbreaks and their associated characteristics was aimed to be provided.
